# MR imaging features of Lhermitte–Duclos disease: Case reports and literature review

**DOI:** 10.1097/MD.0000000000028667

**Published:** 2022-01-28

**Authors:** Han-wen Zhang, Yuan-qing Zhang, Xiao-lei Liu, Yong-qian Mo, Yi Lei, Fan Lin, Yu-ning Feng

**Affiliations:** a Department of Radiology, The First Affiliated Hospital of Shenzhen University, Health Science Center, Shenzhen Second People's Hospital, 3002 SunGangXi Road, Shenzhen, China; b Special Clinic, Shenzhen Children's Hospital, Shenzhen, YiTian Road, China.

**Keywords:** Lhermitte–Duclos disease, MRI, MRS, posterior fossa, PWI, SWI

## Abstract

**Rationale:**

Lhermitte–Duclos disease (LDD) is a rare tumor of the nervous system with a typical “tiger striped’” sign, but its features on functional magnetic resonance imaging (fMRI) are still inconclusive.

**Patient concerns:**

To explore the characteristics of LDDs using fMRI.

**Diagnoses:**

We report 3 cases of pathologically confirmed LDDs.

**Interventions:**

Three patients underwent brain tumor surgery.

**Outcomes:**

All the patients had a good prognosis.

**Lessons:**

Magnetic resonance spectroscopy and susceptibility-weighted imaging combined with conventional MRI can be used to better diagnose LDDs. Perfusion-weighted imaging is not specific for distinguishing cerebellar tumors. The combined application of fMRI and conventional MRI can improve the accuracy of LDD diagnoses.

## 1 Introduction

Dysplastic cerebellar gangliocytoma (also called Lhermitte-Duclos disease [LDD]) is a rare cerebellar tumor composed of dysplastic ganglion cells. Owing to its insidious onset, slow progression, and good prognosis, it is classified as a mixed neuron-glial tumor of WHO class I in the 2016 version of the central nervous system classification.^[1]^ At present, there are still controversies regarding the pathogenesis, genetic characteristics, and even the nature of the tumor (whether it is a true tumor or hamartoma). Loss of the phosphatase and tension homologue (PTEN) gene, located on chromosome ten, is also associated with Cowden syndrome in LDD patients. Some scholars classify LDD as part of Cowden syndrome (multiple hamartoma syndrome).^[2]^

**Figure 1:**
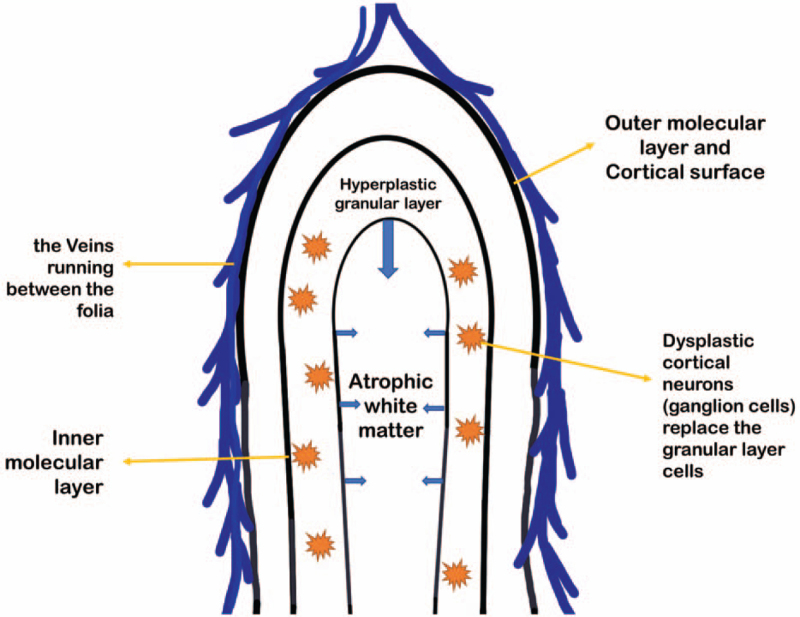
Schematic diagram of the pathology of Lhermitte-Duclos disease.

Currently, the disease is easily pathologically diagnosed as ganglion cell glioma or neuron-glioma.^[3]^ Imaging analysis usually reveals that the normal granular layer cells in the cerebellar lobe are replaced by dysplastic cortical neurones (ganglion cells), resulting in enlargement of the cerebellar lobe. The molecular layer of the brain lobe thickens and produces excessive myelination changes, which lead to layered changes on imaging (Fig. 1).^[4]^ Therefore, for disease confirmation, a typical unk tiger striped’ sign on magnetic resonance imaging (MRI) is required. Therefore, imaging is of great significance for confirming a disease diagnosis.^[5]^

LDD is a rare type of tumor, and most previous studies were case reports. As functional imaging (e.g. perfusion-weighted imaging [PWI], susceptibility-weighted imaging [SWI], and magnetic resonance spectroscopy [MRS]) has been widely used in the clinic, the understanding of the disease has gradually deepened. This article will combine the cases from our center and summaries previous studies to discuss the imaging characteristics of LDDs in different ways.

## 2 Case presentation

A total of 3 LDD patients were admitted to our centre between 2017 and 2021. All patients were aged between 37 and 44 years. In addition to plain MRI and enhanced MRI, some patients underwent related functional imaging examinations, including PWI, SWI, and MRS.

### 2.1 Case 1

The patient was a 40-year-old female. One year prior, she presented with intermittent headaches and neck discomfort of no obvious cause. Assuming cervical spondylosis, rehabilitation physiotherapy was administered, but no obvious improvement was observed. Subsequently, the patient's headache symptoms worsened, accompanied by unstable walking. Physical examination and brain CT suggested the presence of tumorous lesions in the right cerebellum, hydrocephalus, and foramen magnum herniation, and the patient was referred to our hospital. The right fingernose test on the right side of our hospital was positive, and the stance with closed eyes test was positive.

MRI examination in our hospital (Fig. 1) showed a space-occupying lesion in the patient's right cerebellum, and the fourth ventricle was compressed and narrowed. Overall, the lesion showed a hypointense signal on T1-weighted imaging (T1WI), with stripes and other slightly hyperintense signals; on T2-weighted imaging (T2WI), the lesion showed an overall hyperintense signal, with stripes and other hypointense signals, showing a typical “tiger pattern” manifestation. The whole lesion showed limited spread on diffusion-weighted imaging (DWI). There was no obvious enhancement in the whole lesion on the enhanced scan, but scattered, vascular-like enhancement was observed inside. Imaging suggested a typical diagnosis of “LDD”.

The patient underwent resection of the right cerebellar space-occupying lesion under general anesthesia, and the operation progressed smoothly.

Pathological results, light microscopy: glial cell proliferation mixed with a large number of ganglion cell-like cells, edema, formation of microcapsules, and a large number of irregular blood vessels. Immunohistochemical results: GFAP(+), IDH1 R132H(−), Ki67 approximately 1%(+), NeuN(+), Olig-2 part(+), P53(−), Syn(+), CD34(−), CgA(+), NF(+). Final pathological result: “LDD” in the right cerebellar hemisphere.

Anti-inflammatory, intracranial pressure-lowering, and other drugs were administered postoperatively, and the patient's discomfort was treated symptomatically. The patient was regularly reviewed after discharge, and she had a good prognosis within 2 years

### 2.2 Case 2

This patient was a 44-year-old woman. Chronic course, unstable walking for 3 years PET-CT in another hospital showed slight low-density occupancy in the right cerebellar area. PET revealed increased radioactive uptake, suggesting the possibility of malignant tumors. On admission to our hospital, physical examination yielded a limb muscle strength of grade 5, muscle tension, and difficulty in establishing a positive sign with closed eyes; the remaining results were unremarkable. Routine blood test + nucleated red blood cells: red blood cell count 2.69 × 10^12^/L↓, hemoglobin 58.0 g/L↓, average red blood cell volume 82.2 fL, average red blood cell hemoglobin content 21.6 pg↓, average red blood cell hemoglobin concentration 262.0 g/L↓. Clinical considerations of iron-deficiency anemia red blood cell transfusion was performed to improve anemia and platelets were administered to improve blood coagulation function.

MRI at our hospital (Fig. 3) revealed lesions occupying the right cerebellar hemisphere. T1WI showed an overall slightly hypointense signal, T2WI showed an overall slightly hyperintense signal, and the lesions had typical “tiger pattern.” A vascular-like enhanced area can be seen on the enhanced scan. Local vascular-like areas with slightly higher perfusion were observed on PWI. The Cho peak on MRS (Fig. 4) was slightly elevated and the NAA peak was slightly depressed. The Cho/NAA ratio was less than 1, and the Lac peak was observed. A typical diagnosis of LDD was made.

**Figure 2:**
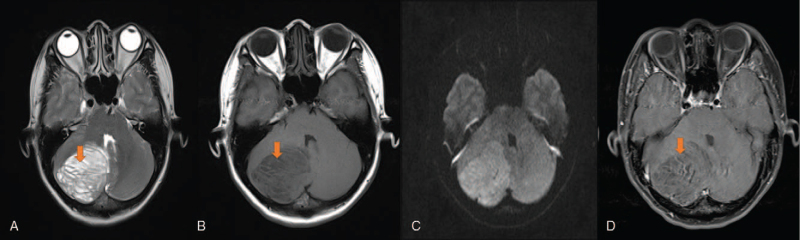
A 40-year-old female patient with intermittent headache. MR examination: A. T2-weighted imaging (orange arrow, tiger striped sign), B. T1-weighted imaging (orange arrow, tiger striped sign), C. enhanced MRI scan showed limited diffusion, D. enhanced MRI scan (orange arrow, vascular-like enhancement).

**Figure 3:**
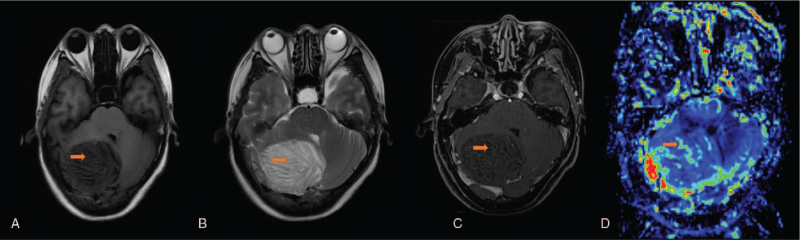
A 44-year-old female patient with occipital headache and unsteady gait. MR examination: A. T1-weighted imaging (orange arrow, tiger striped sign), B. T2-weighted imaging (orange arrow, tiger striped sign), C. Enhanced MRI scan (orange arrow, vascular-like enhancement), D. Perfusion-weighted imaging (increased local perfusion of the lesion).

**Figure 4:**
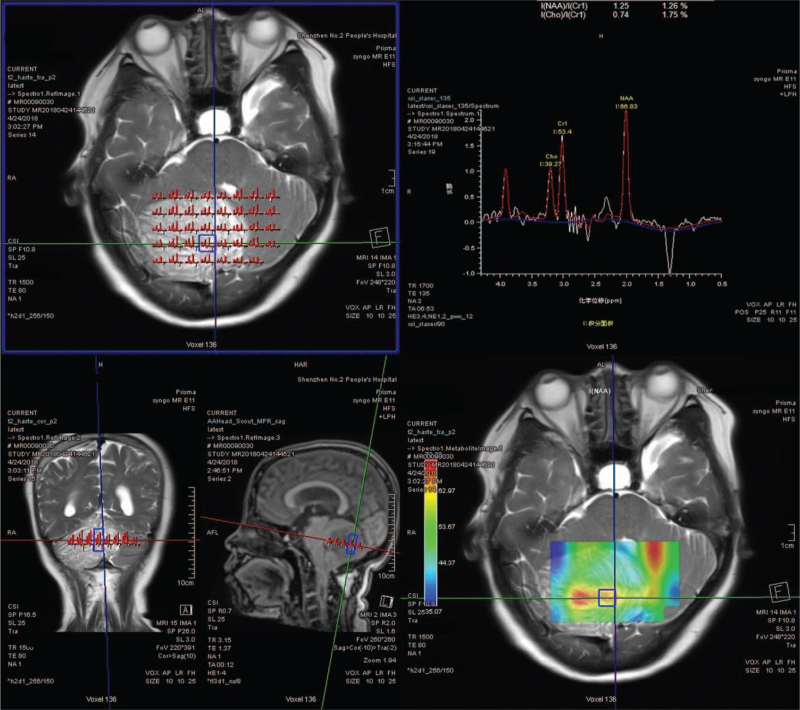
Same patient as in Figure 3. MRS measurement chart.

The patient underwent resection of the right cerebellar space-occupying lesion under general anesthesia, and the operation progressed smoothly.

Pathological results and light microscopy showed that the granular layer of the cerebellum was absent, large and irregular ganglion cell-like nerve cells were seen in a layered distribution, nuclear divisions were difficult to observe, no necrosis was observed, and the molecular layer was thickened. Immunohistochemistry: GFAP (partial +), IDH1 R132H (−), Ki67 (<1% +), CD34 (partial +), NeuN (ganglion cell-like nerve cell +). The pathological results were consistent with the LDD-like manifestations.

The prognosis of the patient was good, and the patient has been regularly reviewed for more than 2 years after the operation. The patient's condition was stable, and there was no recurrence.

### 2.3 Case 3

The patient, male, 37 years old, had dizziness and headache for more than 10 days. Activated partial thromboplastin time 44.4 second↑; blood biochemistry: total calcium 2.03 mmol/L↓. The remaining test results showed no clear abnormalities.

**Figure 5:**
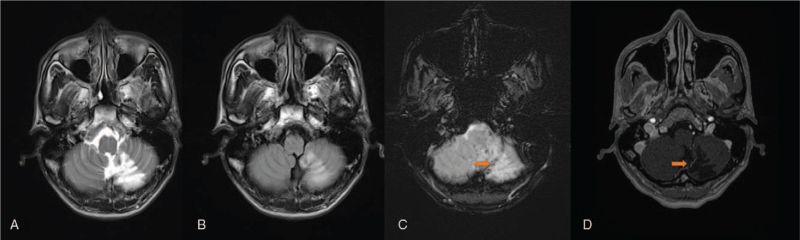
A 37-year-old male patient with headache. A. T2-weighted imaging, B. T1-weighted imaging, C. Susceptibility-weighted imaging (orange arrow, the blood vessels between the brain lobes are clearly displayed), D. Enhanced MRI scan (orange arrow, vascular-like enhancement).

MRI at our hospital (Fig. 5) showed space-occupying lesions in the left cerebellar hemisphere. The entire lesion on T1WI showed isointensity with a slightly hypointense signal on the strips. T2WI showed a hyperintense signal in the entire lesion and a slightly hypointense signal in the internal “tiger pattern” An enhanced scan showed a vascular-like enhancement. SWI revealed abnormally thickened blood vessels in the lesion area. Imaging was used to prioritize the diagnosis of a small dysplastic ganglioma.

The patient underwent total resection of the left cerebellum under general anesthesia. The surgery proceeded smoothly.

Pathological results and light microscopy: The cerebellar tissue structure was disordered, with scattered mature ganglion cells of different sizes. Immunohistochemical results: NF +/−, Syn +, S-100 −, CgA +, GFAP +/−, Ki67 1%, EMA −. Pathological results: LDD.

The patient had a good prognosis within ten months and was followed up regularly.

## 3 Discussion

LDD is a rare benign neoplastic lesion. It is generally believed that the highest prevalence is among individuals aged approximately 30 to 50 years, but its pathogenesis and possible genetic associations remain unclear.^[6]^ Generally, patients are admitted to the hospital because of obstructive hydrocephalus and a sub-tonsillar hernia caused by the excessive size of the lesion, but resection can offer a cure. However, with the gradual application of imaging technology, some reports have advanced the age of onset of this disease to 24 weeks, and the number of reports of disease onset in elderly patients has gradually increased.^[7,8]^ According to a report by Wang et al of West China Hospital in China, 1 patient had tumor recurrence, indicating that LDD may also evolve into a malignant lesion.^[9]^ The gradual application of MRS, SWI, and other technologies in the diagnosis and prognosis of LDD provides valuable information from imaging.

### 3.1 Plain scan and enhanced MRI

All 3 patients with cerebellar dysplastic gangliocytoma in our center had typical “tiger striped” manifestations on MRI.^[10]^ Moreover, on T2WI and FLAIR, this tumor has very unique features. The high-signal areas on T2WI and FLAIR are equivalent to the center of the diseased cerebellar lobe, including the atrophic white matter, a hyperplastic granular cell layer, and a thickened inner molecular layer. The iso-signal area on the MRI was equivalent to that of the brain lobe. On the surface layer, including the outer molecular layer and the pia mater layer (Fig. 1), the enhanced scan usually shows a solid part of the streak enhancement. However, on DWI, the tumor as a whole is usually diffusely limited by water molecules, and the resolution of DWI is usually low; therefore, it does not show unique features.^[11]^

In previous pathological reports, MRI mostly showed round or round-like space-occupying lesions involving one or both cerebellar hemispheres (all 3 patients in our center were affected unilaterally).^[12,13]^ Although the degree of malignancy of this disease is not high, it usually involves a wide range of conditions, which can cause displacement of the fourth ventricle and obstructive hydrocephalus. In almost all cases, conventional MRI can clearly show the “tiger striped” sign of an LDD. The pathology in our center is consistent with that in previous reports (Fig. 2).^[14]^

Although almost all images of LDD patients showed the “tiger striped” sign, some studies reported the appearance of a false “tiger pattern” sign, as reported by Annette C. Douglas-Akinwandea et al, who misdiagnosed medulloblastoma as LDD.^[15]^ Therefore, to improve the diagnostic accuracy, functional imaging other than conventional MRI is necessary.

### 3.2 Perfusion-weighted imaging

According to the literature, 7 patients underwent perfusion-weighted imaging, and 1 patient in our center also underwent PWI.^[16–20]^ We divided the degree of perfusion into high perfusion (+++), local high perfusion (+), and low perfusion (−) (Table 1). We found that 3 patients (37.5%) had obviously high perfusion, 4 (50.0%) had local high perfusion, and 1 (12.5%) had low perfusion.

**Table 1:** Reports of perfusion-weighted imaging on LDD.

Author	Publication date	Sex (age)	Symptoms	Location	Tiger striped	Perfusion
Joachim Klisch, et al	2001 (case 1)	F (49)	Weakness of the limbs Occipital headache Blurred vision	Right cerebellar hemisphere (obstructive hydrocephalus)	Yes	**+**
	2001 (case 2)	M (42)	Occipital headache	Left cerebellar hemisphere	Yes	**+**
B. Thomas, et al	2007 (case 1)	**/**	Loss of pain and temperature sensations Impaired position sense in the lower limbs	Left cerebellar hemisphere	Yes	**+++**
	2007 (case 2)	**/**	Bilateral papilledema, Gaze-evoked nystagmus, Cerebellar signs	Right cerebellar hemisphere	Yes	**+++**
A. Cianfonia, et al	2008	F (46)	Headache	Left cerebellar hemisphere	Yes	**+**
Eve Piekarski, et al	2018	F (62)	Intermittent diplopia	Right cerebellar hemisphere	Yes	**+++**
Cheng, et al	2019	F (48)	Left facial tics Occipital headache Dizziness	Left cerebellar hemisphere	Yes	**–**
Zhang, et al	2022	F (44)	Occipital headache Unsteady gait	Right cerebellar hemisphere	Yes	**+**

PS: Degree of perfusion: high perfusion (+++), local high perfusion (+), low perfusion (-); M = male, F = female, / = unknown.

Although LDD is a tumor with a relatively low malignant potential (WHO I), it can still cause high perfusion on PWI. Based on the analysis of the above cases, we initially believed that, in addition to the local hyperperfusion caused by the blood vessels around the lesion (Fig. 3), the tumor itself could still invade the blood-brain barrier, resulting in an increase in overall tumor perfusion. Cerebellar tumors, including pilocytic astrocytoma and medulloblastoma, all show high perfusion on PWI.^[21,22]^ Nevertheless, the use of perfusion-weighted imaging to obtain a threshold for diagnosing LDD requires additional confirmation.

### 3.3 Magnetic resonance spectroscopy

According to the literature, 9 patients underwent perfusion-weighted imaging, and 1 patient in our center also underwent PWI (Fig. 4).^[16,20,23–28]^ We divided patients according to the presence or absence of the Lac peak (presented as ^∗^) and the Cho/NAA ratio as follows: <1, 1 <ratio <2, and > 2. In total, 7 patients (70.0%) had a ratio <1, 3 (30.0%) had a ratio between 1 and 2, and 0 (0%) had a ratio>2 (Table 2). The Lac peak was observed in all patients.

**Table 2:** Reports of magnetic resonance spectroscopy on LDD.

Author	Publication date	Sex (age)	Symptoms	Location	Tiger striped	Cho/NAA Lac (^∗^)
Joachim Klisch, et al	2001 (case 1)	F (49)	Weakness of the limbs Occipital headache Blurred vision	Right cerebellar hemisphere (obstructive hydrocephalus)	Yes	0.89^∗^
	2001 (case 2)	M (42)	Occipital headache	Left cerebellar hemisphere	Yes	1.02^∗^
Madakasira, et al	2004	F (30)	Blurred vision Occipital headaches	Left cerebellar hemisphere	Yes	<1^∗^
Anik, et al	2007	F (54)	Bilateral papilledema, Gaze-evoked nystagmus, Cerebellar signs.	Right cerebellar hemisphere	Yes	1.28^∗^
Christoph Moenningho, et al	2010	M (46)	Mild gait ataxia, Undirected vertigo	Left cerebellar hemisphere	Yes	<1^∗^
Gioegianni, et al	2013	F (31)	Headache	Bilateral cerebellar hemispheres	Yes	0.7^∗^
Christian Fauria-Robinson, et al	2014	M (71)	Unsteady gait	Right cerebellar hemisphere	Yes	<1^∗^
Pandey, et al	2018	F (29)	Headache	Right cerebellar hemisphere		1<ratio<2^∗^
Cheng, et al	2019	F (48)	Left facial tics Occipital headache Dizziness	Left cerebellar hemisphere	Yes	<1^∗^
Zhang, et al	2022	F (44)	Occipital headache Unsteady walking	Right cerebellar hemisphere	Yes	0.80^∗^

PS: Degree of perfusion, M = male, F = female.

The Cho/NAA ratio is usually used when judging the degree of malignancy of tumors in the brain. An increase in the Cho peak generally indicates that the cell membrane is renewed quickly and the cell density is high. A decrease in NAA usually reflects neuronal loss. In malignant brain tumors, such as high-grade gliomas, on MRS, the Cho peak is significantly high, the NAA peak is significantly low, and the Cho/NAA ratio is high.^[29]^ By analyzing the reported MRS images of LDD patients, we found that the Cho/NAA ratio is usually low, which proves that the tumor is low in malignancy and can be used to differentiate malignant cerebellar tumors, such as medulloblastoma. Moreover, we found that all patients had Lac peak. The Lac peak indicates that anaerobic metabolism of the tissue occurs mostly in malignant tumors. The appearance of the Lac peak, Cho/NAA ratio, and comprehensive presence of the conventional MRI trigger striped sign can improve the diagnostic accuracy of LDD.

### 3.4 Susceptibility-weighted imaging

According to the literature, only 3 patients underwent SWI (1 from our center).^[17,20]^ In fact, the application of SWI in LDD is mainly focused on the anatomical structure—the display of the veins running between the folia (enhancement can also be observed initially, and the interlobular veins are thickened in some cases; Fig. 5). Current cases have reported this phenomenon. The appearance of blood vessels in such lesions is very specific, and low-grade tumors generally have a weak invasion ability and do not cause obvious damage to these blood vessels. As the surrounding normal brain tissue is replaced by dysplastic neurons, the LDD causes these blood vessels to appear more clearly.

LDD usually appears large on conventional MRI and has a typical “tiger striped” sign. Pathology also showed the corresponding components. The diagnosis is usually clear. However, some diseases can also show a false trigger pattern, which requires auxiliary diagnosis with functional imaging. According to existing studies, MRS exhibits a characteristic Lac peak. When the Cho/NAA ratio is low, SWI can reveal thickening of the interlobular veins in the tumor. LDD can be more clearly diagnosed when combined with the unk tiger pattern’ sign. PWI of cerebellar tumors is usually sensitive, but its specificity is low. For PWI to distinguish between these tumors and determine whether the level of LDD perfusion affects the prognosis and recurrence of patients, more patients need to be followed up and analyzed. In non-MR PET, there is a moderate increase in LDD metabolism.^[30]^ However, in some new MRI technologies, such as amide proton transfer (APT) and myelin water imaging, there are no relevant reports on LDDs.^[31,32]^

We found that MRS and SWI are more sensitive for the diagnosis of LDD, while PWI technology requires more cases and literature reports to confirm.

In conclusion, LDD has the characteristics of both conventional and functional imaging. With a more complete examination, we will learn more about this disease, develop more targeted treatments, and provide a better prognosis.

## Acknowledgments

We thank Ms. Siling Gu for her help with this research.

## Author contributions

**Project administration:** Yi Lei.

**Resources:** Yuan-qing Zhang.

**Software:** Fan Lin, Xiao-lei Liu.

**Supervision:** Xiao-lei liu, Yong-qian Mo.

**Writing – original draft:** Hanwen Zhang.

**Writing – review & editing:** Yu-ning Feng.
